# Liquid biopsy in gastric cancer: predictive and prognostic biomarkers

**DOI:** 10.1038/s41419-022-05350-2

**Published:** 2022-10-27

**Authors:** Zihao Zhang, Hao Wu, Wei Chong, Liang Shang, Changqing Jing, Leping Li

**Affiliations:** 1 Department of Gastrointestinal Surgery, Shandong Provincial Hospital, Shandong University, Jinan, Shandong 250021 China; 2 Department of Gastrointestinal Surgery, Shandong Provincial Hospital Affiliated to Shandong First Medical University, Jinan, Shandong 250021 China; 3 Key Laboratory of Engineering of Shandong Province, Shandong Provincial Hospital, Jinan, Shandong 250021 China; 4 Medical Science and Technology Innovation Center, Shandong First Medical University & Shandong Academy of Medical Sciences, Shandong, 250021 China; 5 Department of General Surgery, Peking Union Medical College, Peking Union Medical College Hospital, Chinese Academy of Medical Sciences, Beijing, China

**Keywords:** Gastric cancer, Predictive markers, Diagnostic markers

## Abstract

Gastric cancer (GC) is a high-incidence cancer worldwide. Most patients are diagnosed at an advanced stage, by which time they have limited treatment options and poor prognosis. Early diagnosis and precise treatment are important. In the past few years, emerging research has been conducted on the use of non-invasive liquid biopsy, with its advantages of minimal invasiveness and repeated sampling, to monitor tumor occurrence and recurrence in real time and to evaluate prognosis and treatment response. Many studies have demonstrated the potential of liquid biopsy in GC, and the detection of circulating tumor cells (CTCs), circulating tumor DNA (ctDNA), circulating free DNA (cfDNA), and exosomes has achieved gratifying results. In this review, we summarize evolving technologies for and information regarding liquid biopsy, the most recently discovered GC liquid biopsy biomarkers, and ongoing clinical trials and discuss the challenges and application prospects of liquid biopsy in GC.

## Facts

1. Liquid biopsies, including circulating tumor cells, circulating tumor DNA, and exosomal RNAs, are novel targets for cancer diagnosis, prognosis, and therapy monitoring.
2. Liquid biopsies achieve higher sensitivity and specificity in cancer diagnosis and prognosis by detecting surface biomarkers, DNA methylation, and chromosomal abbreviation.
3. Clinical trials on liquid biopsy for gastric cancer have been carried out, and progress has been made.

## Open questions

1. What is the clinical standard for applying liquid biopsies to gastric cancer patients?
2. What are the most efficient targets for gastric cancer diagnosis, prognosis, and therapy monitoring?
3. Will there be new testing techniques and targets to make liquid biopsy more convenient, economical, and accurate?

## Introduction

**Fig. 1:**
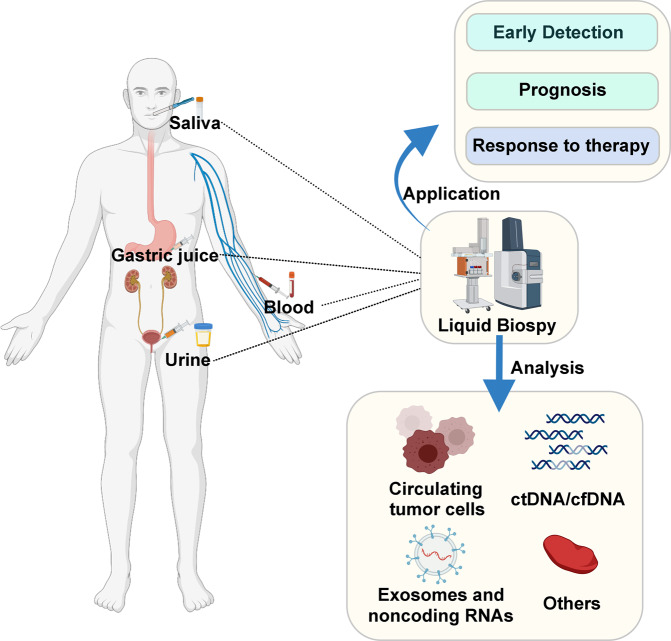
Flowchart of applying liquid biopsy in GC. Blood, saliva, urine, and gastric juice are collected and targets of liquid biopsies, such as CTCs, ctDNA/cfDNA or exosome RNAs, were enriched to achieve early detection, prognosis, and monitoring therapy responses.

Gastric cancer (GC) is one of the most common cancers and represents the third leading cause of cancer death worldwide, and more than one million patients are diagnosed with GC globally each year [1]. Currently, the primary therapy for GC patients is surgery and systemic chemotherapy; in addition, radiotherapy, immunotherapy, and targeted therapy are gradually being used [2], but the 5-year survival rate of GC patients is still unsatisfactory. Therefore, early detection of GC plays a vital role in the treatment and prognosis of GC [3]. The diagnosis of GC is mainly based on imaging and pathological biopsy [4]. However, imaging cannot be used for real-time monitoring of tumors and exposes patients to radiation, and pathological biopsy is an invasive test that causes discomfort for patients. In contrast, liquid biopsy is being increasingly recognized as a tool for GC diagnosis, treatment, and real-time monitoring [5] (Fig. 1). To a certain extent, liquid biopsy, as a non-invasive detection method, can replace traditional invasive physical biopsy. Despite its many advantages, there are also certain limitations and difficulties in its analysis. Therefore, a new technique with high sensitivity is necessary for people with GC who are at high risk of adverse outcomes.

In recent years, several articles have provided a detailed summary of the role of liquid biopsy in several cancers, such as hepatocellular carcinoma (HCC) [6], pancreatic cancer [7], non-small cell lung cancer [8] and melanoma [9], which has caused an increase in liquid biopsy research in the field of cancer. However, in the field of GC, the research progress of liquid biopsy is relatively slow, and no review articles of sufficient quality have been published. Although the study of liquid biopsy related to GC is still in the early stages, the quantity and quality of related research have increased recently. In this review, we mainly emphasize the major advancements in the use of liquid biopsy for early detection, prognostication, and therapy response monitoring in GC.

## Components and applications of liquid biopsy

### Circulating tumor cells (CTCs)

**Fig. 2:**
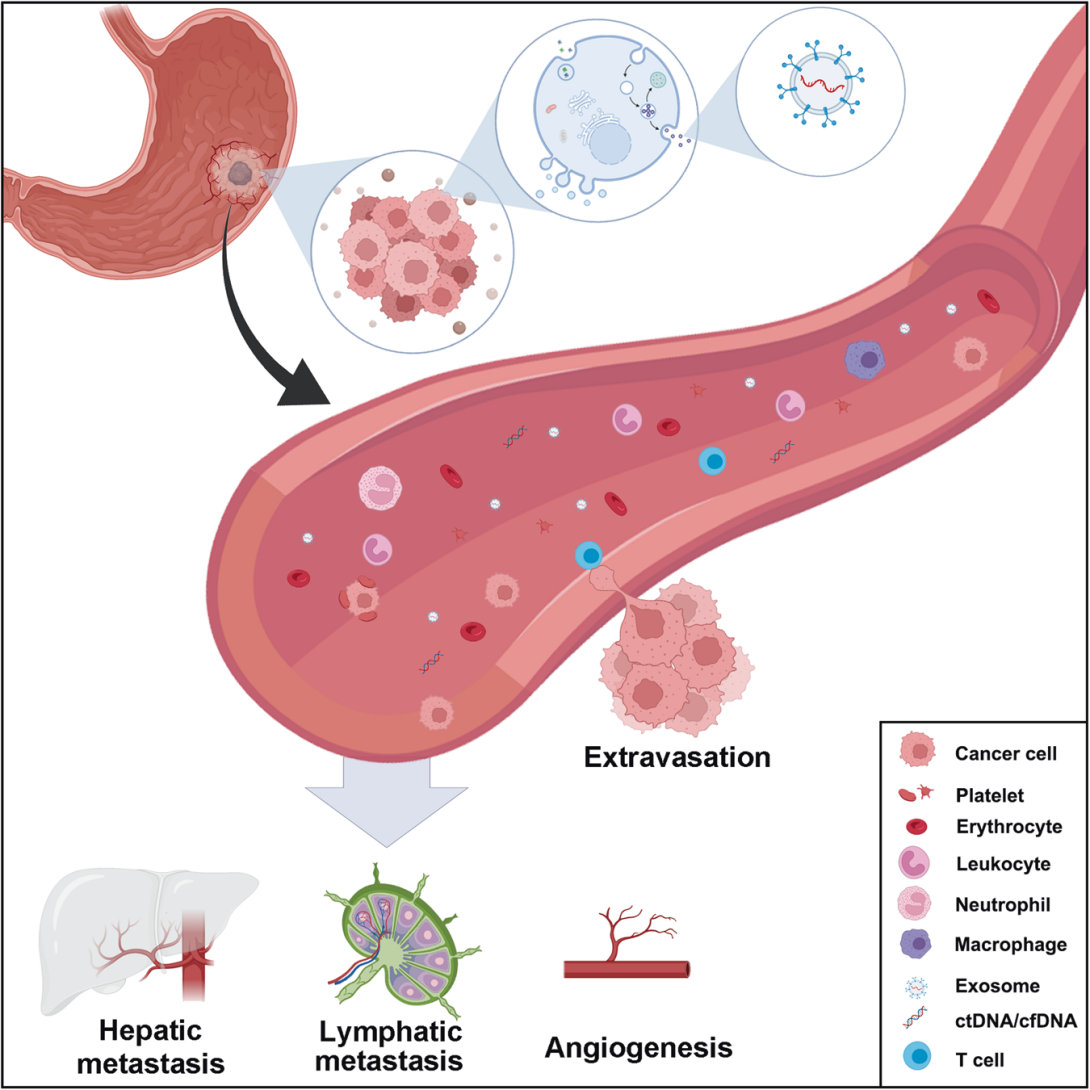
Components of liquid biopsy in GC. CTCs, ctDNA, and exosomes were discovered in the peripheral blood. CTCs and ctDNA are important ingredients that are usually regarded as the foundations of liquid biopsy. ctDNA is formed from apoptotic and necrotic tumor cells, which release fragmented DNA into the bloodstream and possess genetic aberration from original tumor cells. CTCs are cancer cells that spontaneously shed from primary or metastatic tumors and circulate in the circulation. They are tumor “seeds” and can result in recurrence by hepatic metastasis, lymphatic metastasis, and angiogenesis.

Generally, CTCs are cells that are shed from primary tumor foci into the peripheral circulation or invade blood vessels through epithelial−mesenchymal transition [10] (Fig. 2). Thus, CTCs have drawn much attention as valuable diagnostic, prognostic, and even treatment response and resistance biomarkers for various cancers [11]. These cells hold valuable information (Fig. 1). The application of CTCs as prognostic biomarkers has received considerable attention in multiple tumors. First, the number of CTCs is measured. It has been reported that CTCs can be used to predict the survival rate and monitor recurrence after surgery. For example, *Pierga* et al. showed that breast cancer patients with more than one CTC per 7.5 ml of blood had shorter progression-free survival (PFS) (*p* < 0.0001), and those with more than five CTCs per 7.5 ml of blood had shorter overall survival (OS) [12]. In addition to the quantitation of CTCs, chromosome rearrangements in CTCs can be detected by fluorescence in situ hybridization (FISH), genetic changes in CTCs can be detected by genome sequencing, and CTC protein expression can be detected by immunocytochemistry [13]. Based on the characteristics of CTCs, clinicians can select the most appropriate treatment, and therapy response can be reflected in changes in CTCs. In colorectal cancer (CRC), molecular analysis of single CTCs revealed significant intra-patient and inter-patient heterogeneity in mutant *EGFR* expression as well as that of other genetic mutations relating to EGFR inhibition, such as *KRAS* and *PIK3CA* mutations, which explains the diverse response rates to EGFR-targeted therapy in patients with CRC [14]. For patients with castration-resistant prostate cancer, the number of posttreatment CTCs was revealed to be an earlier biomarker to evaluate the effectiveness of treatment than prostate specific antigen (PSA) [15]. Moreover, enriched CTCs can be cultured ex vivo, enabling personalized therapy to be determined based on genetic profiling and novel drug sensitivity testing on ex vivo CTCs [16]. More clinical studies and advanced CTC detection and collection techniques need to be developed to realize the transition of CTC applications from bench to bedside.

#### GC diagnosis through CTCs and cell surface biomarkers

Owing to the malignancy and late diagnosis of GC, early detection of GC is of great value to achieve successful treatment for patients. Only a few studies have focused on applying CTCs as a diagnostic marker for GC; however, the outcomes have been quite surprising (Table 1). According to Yoon-Kyung Cho, 7.5 ml of blood was collected from 115 GC patients and 31 healthy controls, and CTCs were separated via a centrifugal microfluidic system. A total of 97.1% of subjects with more than two CTCs were GC patients, representing a specificity of 90.3%. Nevertheless, 38% of GC patients had less than two CTCs per 7.5 ml, indicating that the sensitivity of this method needs to be improved [17]. According to a previous study, a GC stem cell biomarker, CD44, was specifically expressed in GC patients compared to healthy controls. Thus, diagnosing GC based on CTCs with a high level of CD44 greatly reduced the false-negative rate. The sensitivity of this method reached 92.3% [18]. A recent study demonstrated that protein tyrosine kinase 7 (PTK7), a critical membrane receptor that was first discovered in colon carcinoma cell lines, improved the sensitivity of detecting CTCs in gastric cell line samples combined with epithelial cell adhesion molecule (EpCAM). Nevertheless, more research needs to be done to examine the efficiency of PTK7 and EpCAM in blood samples [19]. In addition, according to Huang et al., CTCs are significantly related to clinicopathological features, such as the differentiation and stage, of tumors [20]. Generally, CTCs are detected by EpCAM on cell surface. However, in GC, some of CTCs with highly invasive and metastatic capacity do not express EpCAM on cell surface. RUBYchip^TM^, a recently developed CTCs isolation technique, recognizes CTCs according to cell size and deformability, greatly improving CTCs capture rates [21].

**Table 1:** Liquid biopsy of CTCs and ctDNA/cfDNA in GC.

Clinical scenario	Liquid biopsy strategy	Study	Target	Patients	Sensitivity	Specificity	AUC
Early detection					Performance		
	CTC detection	[53]	Detection of ≥2 CTCs/7.5 ml blood	116 GC, 31 healthy	85.3%	90.3%	
	CTC marker	[54]	CD44	26 GC, 12 healthy	92.3%	100%	0.9744
	cfDNA	[58]	Detection of cfDNA ≥ 90 ng/ml	30 GC, 34 healthy	96.67%	94.11%	0.991
	ctDNA methylation	[60]	Differentially methylated regions (DMR)	300 GI tumor	/	/	0.90
	ctDNA methylation	[62–65]	XAF1, PCDH10, RASSF1A, RUNX3, RPRML				
Prognosis				Performance			
	CTC detection	[73]	Detection of≥1 CTCs/7.5 ml blood	Association with overall survival and disease-free survival			
	CTC detection	[74]	Detection of ≥5 CTCs/7.5 ml blood	Association with relapse free survival			
	CTC detection	[76]	The number of CTCs after surgery	Association with recurrence and relapse free survival			
	CTC marker	[77]	CEA, EpCAM	Association with shorter three-year relapse free survival			
	CTC marker	[78]	CD44	Association with shorter overall survival			
	CTC marker	[79]	CD133	Association with shorter overall survival			
	CTC marker	[80]	TWIST	TWIST (+) CTCs > 2.5/7.5 mL of blood tended have shorter overall survival			
	Multiploid CTC	[81]	Aneuploidy of chromosome 8	Association with shorter progression-free survival and overall survival			
	ctDNA detection	[84]	Detection of ctDNA after surgery	Association with shorter disease-free survival and overall survival			
	ctDNA detection	[87]	Detection of ctDNA after surgery	Association with shorter relapse-free survival and overall survival			
	cfDNA genetic alteration	[88]	Genetic alteration derived from GC	Association with shorter overall survival			
	ctDNA genetic alteration	[89]	BRAF/FGFR2 amplification and ARAF mutation	Association with shorter overall survival			
	ctDNA genetic alteration	[90]	TP53 mutation and MET amplification	Association with shorter overall survival			
	ctDNA methylation	[92]	Methylated TIMP-3	Association with shorter disease-free survival and overall survival			

#### Predicting GC patients’ prognosis through CTCs

Many studies suggest that CTCs are among the most important prognostic biomarkers for many cancers. Significant correlations between CTC counts and patient survival time have been found in many studies. CTCs were strongly related to poor overall survival (OS) in patients with GC, according to the findings of seven investigations involving 579 patients with GC from four countries [22]. However, the standard number of CTCs used as a cut-off varies in different studies. For example, a study of 72 patients found that patients with more than one CTC in blood had shorter disease-free survival (DFS), (*p* = 0.001) and OS (*p* = 0.0007) than patients without CTCs [23]. In contrast, GC patients with more than five CTCs in 7.5 mL of peripheral blood had a worse relapse-free survival (RFS) than those with less than five CTCs [24]. In addition, it was reported that CTCs tended to aggregate, forming circulating tumor microemboli (CTM), which would facilitate cancer metastasis. The more CTCs aggregated, the worse OS would be. The OS of GC patients with CTM2 (made of 2 CTCs) was almost 10 months longer than that of GC patients with CTM3-4 (made of 3-4 CTCs) [25]. Further studies demonstrated that CTCs expressing specific molecules are potential biomarkers for GC patient prognosis. For instance, N-cadherin is a marker related to GC relapse, and patients can be characterized as having different risks of recurrence according to the number of N-cadherin+ CTCs [26]. Likewise, GC patients with more carcinoembryonic antigen positive (CEA) cells were highly likely to experience relapse within three years [27]. CD44 + cells were commonly detected in the blood or bone marrow of patients with distant metastasis, and their detection was associated with shorter OS [28]. CD133 and ABCG2 are biomarkers of cancer stem cells. GC patients with upregulated CD133 + and ABCG2 + CTCs were found to have shorter OS [29]. In addition, GC patients with more than 2.5 TWIST + CTCs per 7.5 ml of blood had shorter OS than patients with fewer than 2.5 TWIST + CTCs [30]. Moreover, Qiu et al. found that CTCs associated with white blood cell clusters (CTC-WBC clusters) were more sensitive markers to predict prognosis for gastric cancer patients. Patients with CTC-WBC clusters had shorter OS than those without CTC-WBC clusters [31]. Moreover, also genomic alterations in CTCs seem to have prognostic value. In particular, the presence of 2 CTCs with aneuploidy of chromosome 8 per 7.5 mL in GC patients correlated with short PFS and OS [32]. In conclusion, CTCs are a meaningful target in GC prognosis and thus deserve further research.

#### Predicting therapy response via CTCs

CTC-positive (CTC+) patients experience shorter PFS and a lower disease control rate (DCR) after therapy than CTC-negative patients [22]. However, the definition of CTC+ still needs to be clarified. According to a prospective study, patients with more than four CTCs at 2 weeks and 4 weeks after starting chemotherapy had lower median PFS and lower median OS than those with less than four CTCs [33]. Another study indicated that after 6 weeks of chemotherapy, the overall response rate (ORR), DCR, and PFS were significantly shorter in patients with more than four CTCs than in those who had fewer CTCs [34]. Nonetheless, other studies showed that the presence of more than five CTCs in the blood is an appropriate cut-off value for predicting therapy response. Patients with five CTCs or more were highly likely to experience progressive disease (PD) and shorter PFS [35]. Moreover, CTCs can be divided into epithelial type, expressing keratins 8, 18, and 19 and EpCAM, and mesenchymal type, expressing vimentin and TWIST, in GC patients. Increased mesenchymal-type CTCs after chemotherapy indicated that patients were overwhelmingly likely to experience PD [36].

#### CTCs for HER2 + gastric cancer

HER2 is a protein commonly upregulated in a portion of GC cases, which are referred to as HER2+ or *HER2*-amplified GC, and trastuzumab is the first-line therapy for HER2 + GC [37]. Due to tumor heterogeneity, HER2 + GC can be misdiagnosed as HER2- GC when the diagnosis is based on tissue biopsy. When this occurs, patients cannot receive effective and timely treatment with trastuzumab. Reported by Mishima et al., an advanced technique named 3D-IF-FISH has higher sensitivity for detecting HER2 + CTCs than traditional tissue biopsy, and patients with HER2+ detected via CTC analysis but HER2- detected via tissue biopsy were beneficial from trastuzumab therapy [38]. According to Matsushita et al., HER2 + CTCs are an independent prognostic marker for GC patients related to favorable OS and PFS, regardless of histological HER2 status [39]. Furthermore, the number of HER2 + CTCs was reduced after trastuzumab therapy, while it rebounded when drug resistance developed. Therefore, the number of HER2 + CTCs is a useful tool for monitoring the effectiveness of trastuzumab therapy [40]. Innate resistance to trastuzumab can result from *PIK3CA* mutation, and acquired resistance can be caused by mutation of *PIK3R1* or *PIK3C3* or mutation of *HER2*. Hence, it is not surprising that HER2 + CTC patients had short PFS and benefited little from trastuzumab therapy [41].

### Cirtulating tumor DNA (ctDNA)/cell-free DNA (cfDNA)

In the bloodstream, cfDNA is DNA fragments released by cells, and cfDNA is composed of (ctDNA, circulating cell-free mitochondrial DNA, and cell-free fetal DNA. ctDNA refers to DNA fragments originating from cancer cells [42]. Studies have confirmed that ctDNA can be secreted from primary tumor cells, metastatic tumor cells and CTCs and actively or passively released by tumor cells undergoing apoptosis or necrosis [43]. In healthy individuals, cfDNA is nearly absent. In cases of malignant tumors, chronic inflammation, and excessive cell death, cfDNA will substantially accumulate. Allele-specific polymerase chain reaction, amplification at lower denaturation temperatures (COLD), emulsion PCR, and massively parallel sequencing are among the technologies being developed for cfDNA analysis [44]. ctDNA has been detected in many types of cancer. Owing to its origin, ctDNA contains all the genetic features, such as the mutations, amplifications, deletions, and/or translocations, that primary and metastatic tumor cells have. Studies have shown that the analysis of cfDNA and/or ctDNA is a more sensitive method for identifying tumor-specific genetic changes than traditional biopsies or the assessment of normal cancer biomarkers used in the clinic. For instance, the detection rate of *FGFR2* amplification was higher with ctDNA analysis than with tissue biopsy because of tumor heterogeneity, which improves the treatment efficiency [45]. Assessing the methylated cfDNA profile through liquid biopsy significantly improves early diagnosis efficiency. To distinguish methylated ctDNA from a large amount of cfDNA derived from normal tissue, Shen et al. developed cell-free methylated DNA immunoprecipitation and high-throughput sequencing (cfMeDIP-seq), which is able to detect methylated cfDNA in only 1–10 ng of DNA. One of the advantages of cfMeDIP-seq is that different types of samples can be used, for example, urine and cerebrospinal fluid [46]. Nuzzo et al. validated the sensitivity of cfMeDIP-seq as a method to diagnose early-stage renal cell carcinoma via patient blood or urine [47]. Currently, the use of non-invasive liquid biopsies to obtain methylated cfDNA has drawn substantial research attention, and studies have been performed on different cancers. For instance, by comparing HCC tissue and normal blood leukocytes, an HCC-specific methylation marker panel was discovered by Xu et al. After validation in cfDNA from a large clinical cohort, ten HCC-specific methylation markers were selected to construct a diagnostic prediction model with high diagnostic specificity (94.3%) and sensitivity (85.7%) [48]. In 2020, a single ctDNA methylation marker, cg10673833, was discovered for diagnosing CRC and showed 86.8% specificity and 89.7% sensitivity [49].

#### GC diagnosis through ctDNA/cfDNA methylation profile

Notably, cfDNA increases as GC progresses. Diagnosing GC through cfDNA achieved 96.67% sensitivity and 94.11% specificity when the cfDNA threshold was 90 ng/ml [50]. Zhong et al. revealed that the diagnostic value of cfDNA was higher than that of traditional biomarkers, such as CA199, CA125, and AFP, as demonstrated by receiver operating characteristic (ROC) analysis [51]. As mentioned above, methylated cfDNA and ctDNA are important research topics. Recently, genome-wide methylation analysis was performed based on the methylation information of 1781 gastrointestinal stromal (GI) tumors and adjacent normal tissues, which was subsequently validated by 300 cfDNA. The results showed that GI cancers could be distinguished by differentially methylated regions obtained from blood samples [52]. Furthermore, a 153 cfDNA methylation biomarker panel including *DOCK10*, *CABIN1*, and *KCNQ5* was identified in a GC patient cohort, providing a novel method to diagnose early-stage gastric cancer [53]. *XAF1* was found to be downregulated in GC patients because of a high percentage of methylation in cancer tissues (83.2%) compared to paracancerous histologically normal tissues (27.2%) and healthy controls (0%). Consistent with the pathological results, a high frequency of *XAF1* methylation was detected in cfDNA of GC patients (69.8%, 141 of 202), and no methylation was detected in cfDNA of 88 healthy controls [54]. Furthermore, studies found elevated methylated tumor suppressor genes in cfDNA, including *PCDH10*, *RASSF1A*, *RUNX3*, and *RPRML*, specifically in blood samples from GC patients, which produced satisfactory sensitivity and specificity, suggesting that methylation of these genes has the potential to be a diagnostic biomarker [55–57]. Moreover, methylated cfDNA and ctDNA can predict the clinicopathological characteristics of tumors. In several studies, GC patients with lymphatic metastasis, distant metastasis, and advanced TNM stage generally had higher levels of *SFRP2* [58], *APC* [59], and *SOX17* [60] methylation in ctDNA. *Ling* et al. found that the levels of abnormally methylated *MINT2* and *THBS1* in cfDNA were positively correlated with peritoneal dissemination and tumor progression [61, 62].

#### Predicting GC patient prognosis via ctDNA/cfDNA mutation and methylation profile

Owing to the short half-life of ctDNA/cfDNA, they are capable of reflecting tumor status in an almost real-time manner, making them potential prognostic biomarkers for GC patients [63]. Relapse occurred in every GC patient who had detectable ctDNA after surgery, and shorter disease free survival (DFS) and OS were observed in these patients [64]. In addition, GC patients with a high level of long fragment cell-free DNA after curative surgery showed worse RFS and OS [65]. Thirty-five GC patients had single-nucleotide variants and copy number alterations in serum ctDNA in a cohort of forty GC patients, confirming that a high proportion (87.5%) of patients had genomic alterations [66]. The cfDNA level was 44 times higher in GC patients than in healthy controls, while the DNA fragment length became shorter as the scale expanded. cfDNA retains somatic mutations derived from GC tissue and is positively correlated with worse survival time [67]. A study by Catenacci et al. demonstrated that most GC patients (77%) have at least one genomic alteration. The results showed that amplification of *BRAF/FGFR2* and mutation of *ARAF* indicated worse survival [68]. Furthermore, a study suggested that GC patients with *TP53* mutation or *MET* amplification had shorter overall survival than those without these alterations [69]. It is worth mentioning that methylation of cfDNA or ctDNA not only aids the timely detection of GC but is also quickly becoming a key tool for predicting patient outcomes; for example, methylation of *PCDH10*, *RASSF1A*, *XAF1*, *SOX17*, and *WIF-1* in cfDNA or ctDNA has shown potential [54, 55, 70]. Generally, hypermethylation of these genes indicates unfavorable outcomes, such as relapse, poor response to therapy, and worse survival time. *TIMP-3* methylation was upregulated in GC tissue and was found to be strongly associated with peritoneal metastasis and TNM stage. Approximately half of the GC patients were found to have methylated *TIMP-3* in preoperative peritoneal washes and serum samples. Patients with higher methylation levels of *TIMP-3* in their body fluids had shorter DFS [71]. It is easy to conclude that the scale and genetic or epigenetic changes of ctDNA/cfDNA are important parameters to predict the prognosis of GC patients.

#### ctDNA/cfDNA predicting therapy efficiency

The amount of cfDNA or ctDNA after therapy predicts the outcomes of patients suffering from different tumors [72]. After treatment, GC patients with PD had higher concentrations of plasma cfDNA/ctDNA over time; for patients with partial response, the concentration of cfDNA/ctDNA increased; cfDNA/ctDNA remained stable in stable disease patients [51, 72]. PD-L1 therapy is approved as primary immunotherapy to treat metastatic GC, but its efficacy in patients varies. The mutational load of cancer, which can be somewhat reflected by ctDNA, was found to be significantly associated with the response to PD-L1 therapy, specifically pembrolizumab. Patients with a higher mutational load in ctDNA before treatment achieved an ORR of 83.3%, while those with a low mutational load achieved an ORR of only 7.7%. Research also found that patients with reduced ctDNA six weeks after therapy had extended PFS [73]. Thus, ctDNA could be regarded as a response and PFS predictor for GC patients. In addition, chromosomal accessibility of circulating CD8 + T cells is a potential PD-1 blockade therapy predictor for GC patients. Shin et al. showed that the openness of the chromatin structure in circulating CD8 + T cells is associated with a good response to anti-PD-1 therapies and a long survival time in GC patients [74]. Chromosomal instability, represented by copy number instability (CNI) in ctDNA, was negatively correlated with the efficacy of treatment. CNI decreased in patients who were sensitive to treatment, while it increased in patients who were resistant to therapy [75]. In conclusion, it is highly valuable to detect mutations and scales of ctDNA before, after, or during treatment in order to evaluate the efficiency of treatments.

#### ctDNA/cfDNA predicting therapy resistance

A study found that patients with mutations in *TGFBR2*, *RHOA*, and *PREX2* were resistant to PD-1 antibody therapy, and these patients had significantly shorter PFS than those who did not. In addition, the results showed that newly generated mutations in *FOXL2* and *RHOA* and copy number variations of *FGFR2* were responsible for acquired resistance to immunotherapy in patients who previously had good responses to PD-1 antibody therapy [76]. Synchronous MET amplification limits the efficacy of *FGFR2* inhibitors for *FGFE2*-amplified GC patients [45]. MET-amplified oesophagogastric cancer with newly generated *KRAS* mutations and *HER2* amplification resisted MET inhibitor therapy [77]. HER2 + cfDNA indicated a good response rate to therapy. However, cooccurring *CCNE1* amplification in cfDNA was associated with HER2-targeted therapy resistance, while newly emerged *HER2* amplification increased sensitivity to HER2-targeted therapy [78]. Genomic alterations caused by therapy are thought to play a pivotal role in chemotherapy resistance. On the other hand, genomic alterations that are already present in the tumor before the start of any treatment could be the determinant of response to therapy.

### Exosomes and noncoding RNAs

Exosomes have drawn much attention in scientific research in the past ten years, and our team also reviewed the role and application of small extracellular vesicles (EVs) in GC [79]. Exosomes are small (30–140 nm) membrane-bound EVs that are secreted by large multivesicular bodies and are released into the extracellular environment through fusion; in addition, they can be detected in blood, urine, cerebrospinal fluid, and other body fluids. Many cell types can release exosomes, such as epithelial cells, haematopoietic cells, neuronal cells, fibroblasts, adipocytes, and tumor cells. Notably, exosomes regulate and participate in physiological and pathological processes [80]. Studies have shown that noncoding RNAs, for example, microRNAs (miRNAs), circular RNAs (circRNAs), and long noncoding RNAs (lncRNAs), which are normally easily degraded, can be packaged into exosomes, which allows them to remain stable in the extracellular environment and transmit signals between cells and tissues. Many academic studies have elucidated that exosomal RNAs are related to epithelial−mesenchymal transition, angiogenesis, the formation of the premetastatic niche, metastasis, the immune response, and therapeutic resistance, indicating that exosomal RNAs play an important role in the initiation and progression of cancer [81]. In 2008, Li et al. demonstrated that the expression of *miR-21* was elevated in patients suffering from breast cancer. Furthermore, breast cancer patients with higher levels of miR-21 tended to have an advanced clinical stage, lymph node metastasis and shorter OS than patients with lower levels of *miR-21*. Therefore, *miR-21* is regarded as a potential prognostic biomarker for breast cancer. Because of the ease with which they can be collected, exosomes and exosomal RNAs have significant advantages as biomarkers in non-invasive liquid biopsies [82]. Sun et al. established a tumor immune infiltration-associated lncRNA signature composed of seven lncRNAs through computational immune and lncRNA profiling analysis based on data from non-small cell lung cancer cell lines and patients. These seven lncRNAs could be used to divide patients into immune hot and immune cold groups. The results revealed that patients in the immune hot group generally had upregulation of immune checkpoint gene expression, which led to longer OS and a good response to immune checkpoint inhibitor therapy, indicating its potential in monitoring therapy response [83].

#### GC diagnosis through exosomal noncoding RNA

In recent years, a considerable amount of literature related to the use of exosomes and exosomal RNAs as diagnostic biomarkers for GC has appeared. In general, in these studies, blood samples were collected from both GC patients and healthy controls, and the level of exosomal RNAs was measured via quantitative reverse transcription PCR. Exosomal RNAs with significant expression differences between GC patients and healthy controls are regarded as candidate exosomal RNAs. Then, the sensitivity, specificity, and area under the ROC curve (AUC) values of these candidate exosomal RNAs were calculated. Those exosomal RNAs with favorable values are reported as potential diagnostic biomarkers. Some studies use a combination of miRNAs to increase the efficiency of diagnosis. For instance, a circRNA panel was identified by Roy et al. based on a cohort of 194 GC patients. This panel achieved the diagnosis of GC patients from healthy controls with high sensitivity and specificity in both the validation and training phases. Furthermore, this circRNA panel was capable of distinguishing early-stage GC from healthy controls, as well as the histological type of GC [84]. In addition, an onco-miRNA panel consisting of exosomal *miR-10a-5p*, *miR-19b-3p*, *miR-215-5p*, and *miR-18a-5p* was also identified [85]. Detailed information on diagnostic exosomal RNAs is presented in Table 2 below.

**Table 2:** Exosomal RNAs in diagnosis of GC.

Biomarker	Type	Expression	Sensitivity/specificity	Correlation	AUC
miR-1290	microRNA	up	26%/90%	TNM stage, distant metastasis	0.657
miR-25	microRNA	up	69.4%/84.6%	TNM stage, LNM	0.768
miR-222	microRNA	up	62.5%/56.2%		0.747
miR-196a	microRNA	up	69.5%/97.6		0.864
miR-196b	microRNA	up	62.2%/96.1%		0.811
miR-26a	microRNA	down	83.6%/81.5%		0.882
miR146-3p	microRNA	down	74.4%/84.1%		0.839
miR-148a	microRNA	down	75.4%/83.1%		0.842
miR-195	microRNA	down	69.2%/75.4%		0.765
miR-106a	microRNA	up	72.9%/63.6%	TNM stage, LNM	0.828
miR-21	microRNA	up	88.4%/79.6%		0.912
miR-421	microRNA	up	90%/85.7%		0.779
miR-199a-3p	microRNA	up	76%/74%		0.818
miR-200c	microRNA	up	65.4%/100%		0.715
miR-212	microRNA	down	95.1%/78.7%		0.960
miR-1, 20a, 27a, 34, 423-5p	microRNA	up	80%/81%	TNM stage	0.879
miR-19a-3p, 483-5p	microRNA	up	87.7%/62.8%		0.84
miR-627, 629, 652	microRNA	up	86.7%/85.5%		0.941
hsa_circ_0000190	circRNA	down	41.4%/97.5%	TNM stage, LNM	0.60
hsa_circ_0130810	circRNA	down	77.42%/68%	TNM stage, LNM	0.748
hsa_circ_0021977	circRNA	down	85.85%/95.24%	TNM stage	0.933
hsa_circ_0006848	circRNA	down	73.3%/90%	TNM stage, tumor differentiation	0.825
hsa_circ_0001821	circRNA	down	86.67%/86.67%	TNM stage, LNM	0.872
hsa_circ_0065149	circRNA	down	48.7%/90.2%	TNM stage	0.640
FRLnc1	lncRNA	up	74.5%/76.8%	TNM stage, LNM	0.709
RP11-731F5.2	lncRNA	up	81.63%/63.64%		0.78
lncRNA-GC1	lncRNA	up	87.21%/87.10%	clinical stage	0.886
CEBPA-AS1	lncRNA	up	87.9%/78.8%	TNM stage	0.824
PCGEM1	lncRNA	up	72.9%/88.9%	TNM stage, tumor differentiation	0.75
HOXA11-AS	lncRNA	up	78.7%/97.8%	TNM stage, LNM	0.924
lncUEGC1	lncRNA	up	88.24%/83.33%	TNM stage	0.876
HOTTIP	lncRNA	up	69.8%/85.0%	TNM stage	0.827
PVT1	lncRNA	up	80.2%/60.4%	TNM stage, LNM	0.728
HULC	lncRNA	up	82%/83.6%	TNM stage	0.888
H19	lncRNA	up	82.9%/72.9%		0.838
UCA1	lncRNA	up		TNM stage, LNM	0.883

#### GC prognosis through exosomal noncoding RNAs

Many studies to determine the prognostic value of exosomal RNAs are underway. Most of these studies focus on the correlation between exosomal RNAs and survival time. Unfortunately, only a few studies have investigated the mechanism behind the correlation. For instance, Fan et al. revealed that there was a close relationship between exosomal *PD-L1* and worse OS in GC patients. The reason for the unfavorable outcomes was that exosomal *PD-L1* exerted suppressive effects on the immune status of GC patients by reducing the number of CD4 + T cells, CD8 + T cells, and granzyme B + cells [86]. Details of exosomal RNAs as prognostic biomarkers are shown in Table 3.

**Table 3:** Exosomal RNAs in prognostic evaluation of GC.

Biomarker	Type	Expression	Correlation
miR-25	microRNA	up	OS, RFS
miR-212	microRNA	down	OS
miR-144	microRNA	down	OS, DFS
miR-215-5p	microRNA	up	OS
miR-3178	microRNA	down	OS
miR-21-3p	microRNA	up	OS, PFS
miR-324-5p	microRNA	up	OS
miR-203	microRNA	down	OS, DFS
hsa_circ_0021977	circRNA	down	OS
hsa_circ_0000936	circRNA	up	OS
hsa_circ_405576	circRNA	up	OS, RFS
hsa_circ_0065149	circRNA	down	OS
RP11-731F5.2	lncRNA	up	OS
HOXA11-AS	lncRNA	up	OS
HOTTIP	lncRNA	up	OS
PVT1	lncRNA	up	OS, DFS

### Tumor-educated platelets

Tumor-educated platelets (TEPs) are one of the newest liquid biopsy components. On the one hand, TEPs help cancer cells grow and escape from the immune system. Platelets exhibiting particular RNA fingerprints from cancer cells are said to have been educated by cancer [87]. As reported by Calverley et al., 197 platelet genes were downregulated in patients with metastatic lung cancer [88]. Moreover, not only platelet genes but also cancer-related genes were expressed in TEPs. Nilsson et al. found that cancer-specific *EGFR* and *PCA3* RNAs were expressed in platelets of glioma and prostate cancer patients [89]. Therefore, TEPs have received considerable attention for their impressive diagnostic value in cancer. In 2015, pan-cancer research on TEPs was performed by Best et al. In terms of mRNA profiles, there were significant differences between TEPs and normal controls in multiple tumor types, and these profiles had 89% accuracy in cancer diagnosis [90].

### Other resources

The most important source of specimens in liquid biopsy is blood. Blood-based biopsy technologies were the earliest to be developed because they can be widely used in clinics and cause relatively little damage to patients. Studies suggest that body fluids other than the blood can also be critical sources for liquid biopsies [91]. For instance, bladder cancer patients could be distinguished from healthy controls by detecting the frequency of *FGFR3* mutations in urine [92]. Head and neck squamous cell carcinomas can be diagnosed by detecting tumor DNA in saliva [93]. Tumor mutations can be found in the cerebrospinal fluid cfDNA of individuals with various primary or metastatic brain cancers [94]. In terms of GC, more studies exploring the application of body fluids other than blood in GC are needed. Nerve growth factors released by GC cells throughout development stimulate salivary glands, resulting in significant changes in the saliva RNA profile that can be utilized to detect GC [95]. Concerning salivary extracellular RNA, a panel comprising three mRNAs and two miRNAs was established and showed high feasibility in diagnosing GC; the panel was also validated in a cohort of 294 individuals [96]. Gastric juice and washes have the closest relationship with GC and are essential resources for liquid biopsies. The differential expression of certain miRNAs and lncRNAs (including *miR-21*, *miR*-*106a*, *miR-129*, and *lncRNA-AA174084*) in gastric juice has the potential to diagnose GC [97–99]. DNA methylation was detected in gastric washes, and GC patients and normal controls showed significant differences in methylation profiles. The sensitivity and specificity reached 90% and 96%, respectively, when diagnosing GC via MINT methylation profiles [100]. Substances secreted by GC cells enter the blood first and are ultimately excreted into the urine through the kidneys. In urine samples, miR-6807-5p, miR-6856-5p, and miR-23-5p were found to be initially highly expressed and reduced after surgery in GC patients, which made them potential biomarkers for early detection and monitoring therapy response [101, 102]. In addition, upregulation of the serum antibody BRAT1 was found to be useful in achieving early detection of gastrointestinal cancers [103].

## Applications of and perspectives on liquid biopsy in GC

### Clinical trials of liquid biopsy in GC

The clinical trials of GC-related liquid biopsy are relatively extensive, as it is a common area of interest for clinicians (Table 4). As listed in the table, the purpose of most liquid biopsies is to predict prognosis and evaluate therapeutic efficiency. Three other trials are aimed at achieving an early diagnosis of GC through liquid biopsies. For example, cfMeDIP-Seq assay is used to not only screen cancer patients from healthy people but also to distinguish cancer types. Tumor-derived mutations, for instance, mutations of *CDH1* and *CTNNA1* which are commonly detected in hereditary diffuse GC, are detected by next-generation sequencing. To achieve prognosis prediction, the majority of clinical trials are designed to detect ctDNA or CTCs before and after GC treatment in a scheduled interval to evaluate the correlation with OS, DFS PFS, and RFS. Concerning therapeutic evaluation, a trial for HER2 positive GC was launched by scholars from China, intending to evaluate the HER2 targeted therapy by monitoring *HER2* status in CTCs, *HER2* amplifications in cfDNA, and therapy resistance-related gene status in both CTCs and cfDNA from before treatment to disease remission or progression. In recent years, immunotherapy research has achieved impressive results in many tumors, including GC. To select GC patients suited for immunotherapy, several clinical trials have evaluated the efficacy of immunotherapy via liquid biopsy. *FoundationOneLiquid*, applied by PLATON, provides information like microsatellite instability (MSI) and tumor mutational burden to help inform immunotherapy decisions. Moreover, for MSI-high gastric/gastroesophageal junction cancer, whether to use tremelimumab and durvalumab as neoadjuvant or definitive treatment is evaluated by clinical trial INFINITY. Another clinical trial, named INTEGA, compared the efficacy between chemotherapy-free immunotherapy and immunotherapy after FOLFOX chemotherapy for HER2 + gastric/gastroesophageal junction cancer patients. Results showed that early cfDNA increases strongly correlated to shorter PFS and OS. Besides, trastuzumab resistance is due to HER2 mutation and epitope scape, resulting in trastuzumab resistance loss. In addition, scholars are evaluating the probability of other body fluids, like gastric juice and peritoneal lavage, being potential liquid biopsy samples for GC.

**Table 4:** Liquid biopsy in clinical trials of GC.

Identifier	Disease	Intervention	Purpose	Country
NCT04253106	Hereditary Diffuse Gastric Cancer	plasma and gastric fluid genetic profiles	Diagnosis	France
NCT04943406	Gastric Cancer/Gastric Adenocarcinoma	plasma and peritoneal lavage ctDNA	Prognosis	Italy
NCT05029869	Gastric Cancer	plasma ctDNA	Prognosis	Vietnam
NCT03957564	Gastric Cancer/Gastro-esophageal Junction Cancer	plasma CTC, ctDNA and cfDNA	Prognosis and response to neoadjuvant chemotherapy	China
NCT04385316	Gastric Cancer	plasma ctDNA	Diagnosis	China
NCT02610218	Advanced/Metastatic Gastric Cancer	plasma CTC and cfDNA	Prognosis and response to targeted therapy	China
NCT04665687	Early Gastric Cancer	plasma ctDNA	Prognosis and diagnosis	Korea
NCT05027347	Early Gastric Cancer	plasma ctDNA	Prognosis	Vietnam
NCT04000425	Gastric Cancer	plasma ctDNA	Prognosis and response to adjuvant chemotherapy	China
NCT04817826	Gastric Cancer	plasma ctDNA	Prognosis and response to neoadjuvant immunotherapy	Italy
NCT04484636	Gastric Cancer	plasma ctDNA	Response to immunotherapy	Germany
NCT03409848	Gastric Cancer	plasma ctDNA	Response to immunotherapy	Germany

### Challenges and perspectives of liquid biopsy in GC

**Fig. 3:**
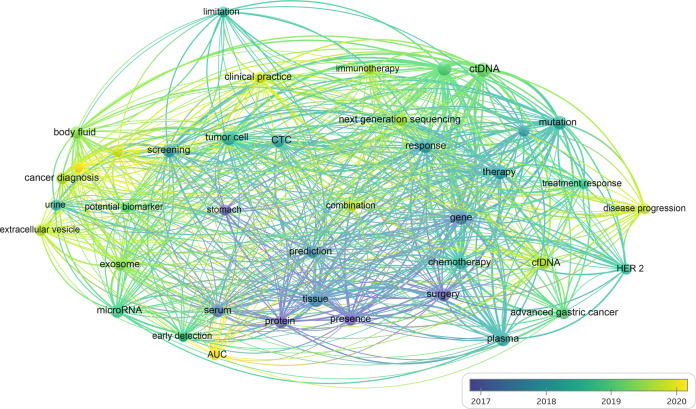
Network and timing analysis based on published literature. Articles published on the Web of Science in the past five years were retrieved and results were visualized through bibliometric methods by VOSviewer and CiteSpace. “Gastric cancer” and “Liquid biopsy” are set as keywords to draw the network and time series diagrams to analyze the development trends in this field. Each node represents different research content. The size of each node indicates the frequency of occurrence, the thickness of the connection between the nodes indicates the strength of the association, and the different color represent the temporal characteristics of the nodes.

**Fig. 4:**
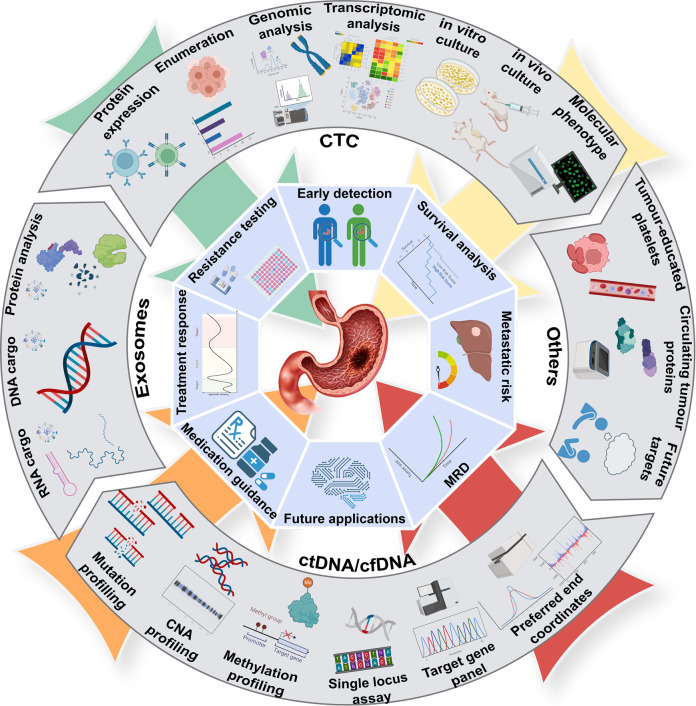
Overview of characteristics and clinical applications of liquid biopsy in gastric cancer. Liquid biopsy is regarded as an important tool in the future, as it could achieve multiple goals, such as early detection, medication guidance, treatment monitoring, resistance testing, minimal residual disease (MRD) detection, survival analysis and metastatic prediction. CTCs and ctDNA/cfDNA are primary targets of liquid biopsy, while other targets are being explored, for example, exosomes containing proteins or DNA and RNA fragments, tumor-educated platelets and circulating tumor proteins. Methods like mutation profiling, CNA profiling, methylation profiling and preferred end coordinates detections are used to acquire qualitative and quantitative information of ctDNA. Assays for single locus and multiple target gene panel are being developed. CTCs provide DNA, RNA protein expression and molecular phenotype at single cell level and can be cultured in vivo or in vitro for further investigation.

Liquid biopsies, especially ctDNA and CTCs testing, have drawn a lot of attention. Minimally invasive sampling, dynamic monitoring, and a comprehensive understanding of cancer are its main advantages. Furthermore, there is sufficient research to suggest that liquid biopsy for early-stage and advanced cancers has implications for clinical decision-making, especially when tissue biopsy is suboptimal or inaccessible. Thus, we performed a search of articles published on the Web of Science in the past five years and visualized the evidence-based results through bibliometric methods. We used “gastric cancer” and “liquid biopsy” as keywords to draw the network and time series diagrams to analyze the development trends in this field (Fig. 3). The application of liquid biopsies shifts from diagnosis and prognosis to treatment management, such as chemotherapy and immunotherapy. The sample changes from blood plasma to other body fluids, such as urine, cerebrospinal fluid, gastric juice, and saliva. The targets develop from protein tumor markers to circulating tumor cells or DNA(CTC/ctDNA) to circulating tumor RNA, exosomal RNA, and tumor-educated platelets. The analysis method shifts from quantification to genomic or epigenomic analysis to in vitro or in vivo experiments (Fig. 4). However, the development and progression of liquid biopsy in GC is relatively slow compared to other cancer, such as lung cancer, colorectal cancer, and hepatocellular cancer. Therefore, liquid biopsy in GC has great potential for making further progress.

In terms of early GC diagnosis, common tumor-driver gene mutations in cfDNA, such as *HER2*, *EGFR*, *RAS*, *TP53*, and epigenetic alternations detected by cfMeDIP-Seq, such as *hMLH*, *CDH1*, *CDKN2A*, *H3K27*, are sensitive target of tumorigenesis, which can be detected when clinical symptoms and imaging test are negative. Hence, a ctDNA assay containing the most frequent genomic aberrations and methylation sites is able to detect early-stage GC in time.

Moreover, liquid biopsy is expected to play a significant role in guiding treatment for GC in three aspects, treatment selection, treatment monitoring, and treatment resistance, as it is in other cancers. First, genetic aberrations that are targeted by certain drugs can be detected by ctDNA. For example, detected by *Cobas EGFR mutation test v2*, a digital PCR assay approved by FDA, non-small-cell lung cancer patients with *EGFR* exon 19 deletion or exon 21 L858R mutation can be treated by EGFR tyrosine kinase inhibitor erlotinib. Besides, PI3Kα inhibitor alpelisib is suitable for breast cancer patients with *PIK3CA* mutations detected by the *PIK3CA RGQ PCR kit*. It is reasonable to foresee commercial assays detecting GC-related genetic mutations, *HER2*, *EGFR*, *KRAS*, etc., being used to help doctors treat patients. Treatment efficiency can be monitored by CTCs and ctDNA after primary treatment, as they represent the tumor burden and minimal residual disease. Once the efficiency of treatment dropped unexpectedly, patients are likely resistant to present treatment. ctDNA could not only detect known resistance mechanisms, such as co-mutations and CNAs, but also unknown resistance mechanisms by large gene panels. Alternative treatments can be tested by CTCs isolated from patients with developing resistance. According to previous research, long-term expansion cells, xenografts, spheroids, and organoids are prepared to overcome drug resistance by mimicking patients’ tumor microenvironment step by step.

With respect to the prognosis for GC, CTCs with specific surface markers deserve further exploration. CTCs detection before treatment can be used as baseline data to stratify patients into different groups. With serial blood testing after primary treatment, the possibility of cancer relapse and metastasis could be predicted.

Although the exploration of liquid biopsy is advancing, the challenges of applying liquid biopsy should not be underestimated. The number of CTCs and ctDNAs is low and their distribution is uneven in peripheral circulation, which greatly limits the reproducibility of liquid biopsy. Besides, the standardization of liquid biopsy needs to be improved, due to the complex procedures, for instance, sample collection, target separation and detection, and lack of reference materials. Additionally, multicentre, larger, and longer-term clinical trials, are urgently required for the clinical use of liquid biopsies.

## Conclusion

In recent years, liquid biopsy has received extensive attention from clinicians. In this article, the components of liquid biopsy and the role they played in GC are discussed in greater detail. With liquid biopsy, early diagnosis, prognosis prediction, and treatment guidance for GC will become easier in the near future, providing new opportunities for management, and even overcoming the GC.

## References

[1] Sung H, Ferlay J, Siegel RL, Laversanne M, Soerjomataram I, Jemal A (2021). Global cancer statistics 2020: GLOBOCAN estimates of incidence and mortality worldwide for 36 cancers in 185 countries. CA Cancer J Clin.

[2] Joshi SS, Badgwell BD (2021). Current treatment and recent progress in gastric cancer. CA Cancer J Clin.

[3] Tan MC, Graham DY (2021). Screening for gastric cancer: focus on the ants instead of the ant hill. Clin Gastroenterol Hepatol.

[4] Smyth EC, Verheij M, Allum W, Cunningham D, Cervantes A, Arnold D (2016). Gastric cancer: ESMO clinical practice guidelines for diagnosis, treatment and follow-up. Ann Oncol.

[5] Salati M, Orsi G, Smyth E, Aprile G, Beretta G, De Vita F (2019). Gastric cancer: translating novels concepts into clinical practice. Cancer Treat Rev.

[6] Ye Q, Ling S, Zheng S, Xu X (2019). Liquid biopsy in hepatocellular carcinoma: circulating tumor cells and circulating tumor DNA. Mol Cancer.

[7] Hou J, Li X, Xie KP (2021). Coupled liquid biopsy and bioinformatics for pancreatic cancer early detection and precision prognostication. Mol Cancer.

[8] Nagasaka M, Uddin MH, Al-Hallak MN, Rahman S, Balasubramanian S, Sukari A (2021). Liquid biopsy for therapy monitoring in early-stage non-small cell lung cancer. Mol Cancer.

[9] Lim SY, Lee JH, Diefenbach RJ, Kefford RF, Rizos H (2018). Liquid biomarkers in melanoma: detection and discovery. Mol Cancer.

[10] Pantel K, Speicher MR (2016). The biology of circulating tumor cells. Oncogene.

[11] Kilgour E, Rothwell DG, Brady G, Dive C (2020). Liquid biopsy-based biomarkers of treatment response and resistance. Cancer Cell.

[12] Pierga JY, Hajage D, Bachelot T, Delaloge S, Brain E, Campone M (2012). High independent prognostic and predictive value of circulating tumor cells compared with serum tumor markers in a large prospective trial in first-line chemotherapy for metastatic breast cancer patients. Ann Oncol.

[13] Sharma S, Zhuang R, Long M, Pavlovic M, Kang Y, Ilyas A (2018). Circulating tumor cell isolation, culture, and downstream molecular analysis. Biotechnol Adv.

[14] Gasch C, Bauernhofer T, Pichler M, Langer-Freitag S, Reeh M, Seifert AM (2013). Heterogeneity of epidermal growth factor receptor status and mutations of KRAS/PIK3CA in circulating tumor cells of patients with colorectal cancer. Clin Chem.

[15] de Bono JS, Scher HI, Montgomery RB, Parker C, Miller MC, Tissing H (2008). Circulating tumor cells predict survival benefit from treatment in metastatic castration-resistant prostate cancer. Clin Cancer Res.

[16] Yu M, Bardia A, Aceto N, Bersani F, Madden MW, Donaldson MC (2014). Cancer therapy. Ex vivo culture of circulating breast tumor cells for individualized testing of drug susceptibility. Science.

[17] Kang HM, Kim GH, Jeon HK, Kim DH, Jeon TY, Park DY (2017). Circulating tumor cells detected by lab-on-a-disc: role in early diagnosis of gastric cancer. PLoS One.

[18] Watanabe T, Okumura T, Hirano K, Yamaguchi T, Sekine S, Nagata T (2017). Circulating tumor cells expressing cancer stem cell marker CD44 as a diagnostic biomarker in patients with gastric cancer. Oncol Lett.

[19] Li C, Yang S, Li R, Gong S, Huang M, Sun Y (2022). Dual-aptamer-targeted immunomagnetic nanoparticles to accurately explore the correlations between circulating tumor cells and gastric cancer. ACS Appl Mater Interfaces.

[20] Huang X, Gao P, Sun J, Chen X, Song Y, Zhao J (2015). Clinicopathological and prognostic significance of circulating tumor cells in patients with gastric cancer: a meta-analysis. Int J Cancer.

[21] Carneiro A, Piairo P, Teixeira A, Ferreira D, Cotton S, Rodrigues C (2022). Discriminating epithelial to mesenchymal transition phenotypes in circulating tumor cells isolated from advanced gastrointestinal cancer patients. Cells.

[22] Yang C, Zou K, Yuan Z, Guo T, Xiong B (2018). Prognostic value of circulating tumor cells detected with the CellSearch System in patients with gastric cancer: evidence from a meta-analysis. Onco Targets Ther.

[23] Qian C, Cai R, Zhang W, Wang J, Hu X, Zhang Y (2021). Neutrophil-lymphocyte ratio and circulating tumor cells counts predict prognosis in gastrointestinal cancer patients. Front Oncol.

[24] Ito H, Sato J, Tsujino Y, Yamaguchi N, Kimura S, Gohda K (2016). Long-term prognostic impact of circulating tumour cells in gastric cancer patients. World J Gastroenterol.

[25] Chen Y, Yuan J, Li Y, Li X, Yang Y, Li J (2021). Profiling heterogenous sizes of circulating tumor microemboli to track therapeutic resistance and prognosis in advanced gastric cancer. Hum Cell.

[26] Ishiguro Y, Sakihama H, Yoshida T, Ichikawa N, Homma S, Fukai M (2021). Prognostic significance of circulating tumor cells with mesenchymal phenotypes in patients with gastric cancer: a prospective study. Ann Surg Oncol.

[27] Miki Y, Yashiro M, Kuroda K, Okuno T, Togano S, Masuda G (2021). Circulating CEA-positive and EpCAM-negative tumor cells might be a predictive biomarker for recurrence in patients with gastric cancer. Cancer Med.

[28] Szczepanik A, Sierzega M, Drabik G, Pituch-Noworolska A, Kołodziejczyk P, Zembala M (2019). CD44(+) cytokeratin-positive tumor cells in blood and bone marrow are associated with poor prognosis of patients with gastric cancer. Gastric Cancer.

[29] Xia P, Song CL, Liu JF, Wang D, Xu XY (2015). Prognostic value of circulating CD133(+) cells in patients with gastric cancer. Cell Prolif.

[30] Jhi JH, Kim GH, Park SJ, Kim DU, Lee MW, Lee BE (2021). Circulating tumor cells and TWIST expression in patients with metastatic gastric cancer: a preliminary study. J Clin Med.

[31] Qiu Y, Zhang X, Deng X, Zhang R, Cai Z, Zhang Z (2021). Circulating tumor cell-associated white blood cell cluster is associated with poor survival of patients with gastric cancer following radical gastrectomy. Eur J Surg Oncol.

[32] Li Y, Zhang X, Gong J, Zhang Q, Gao J, Cao Y (2016). Aneuploidy of chromosome 8 in circulating tumor cells correlates with prognosis in patients with advanced gastric cancer. Chin J Cancer Res.

[33] Matsusaka S, Chìn K, Ogura M, Suenaga M, Shinozaki E, Mishima Y (2010). Circulating tumor cells as a surrogate marker for determining response to chemotherapy in patients with advanced gastric cancer. Cancer Sci.

[34] Li Y, Gong J, Zhang Q, Lu Z, Gao J, Li Y (2016). Dynamic monitoring of circulating tumour cells to evaluate therapeutic efficacy in advanced gastric cancer. Br J Cancer.

[35] Lee SJ, Lee J, Kim ST, Park SH, Park JO, Park YS (2015). Circulating tumor cells are predictive of poor response to chemotherapy in metastatic gastric cancer. Int J Biol Markers.

[36] Li TT, Liu H, Li FP, Hu YF, Mou TY, Lin T (2015). Evaluation of epithelial-mesenchymal transitioned circulating tumor cells in patients with resectable gastric cancer: Relevance to therapy response. World J Gastroenterol.

[37] Smyth EC, Nilsson M, Grabsch HI, van Grieken NC, Lordick F (2020). Gastric cancer. Lancet.

[38] Mishima Y, Matsusaka S, Chin K, Mikuniya M, Minowa S, Takayama T (2017). Detection of HER2 amplification in circulating tumor cells of HER2-negative gastric cancer patients. Target Oncol.

[39] Matsushita D, Uenosono Y, Arigami T, Yanagita S, Okubo K, Kijima T (2021). Clinical significance of circulating tumor cells in the response to trastuzumab for HER2-negative metastatic gastric cancer. Cancer Chemother Pharm.

[40] Wang H, Li B, Liu Z, Gong J, Shao L, Ren J (2018). HER2 copy number of circulating tumour DNA functions as a biomarker to predict and monitor trastuzumab efficacy in advanced gastric cancer. Eur J Cancer.

[41] Wang DS, Liu ZX, Lu YX, Bao H, Wu X, Zeng ZL (2019). Liquid biopsies to track trastuzumab resistance in metastatic HER2-positive gastric cancer. Gut.

[42] Spellman PT, Gray JW (2014). Detecting cancer by monitoring circulating tumor DNA. Nat Med.

[43] Stroun M, Maurice P, Vasioukhin V, Lyautey J, Lederrey C, Lefort F (2000). The origin and mechanism of circulating DNA. Ann NY Acad Sci.

[44] Luke JJ, Oxnard GR, Paweletz CP, Camidge DR, Heymach JV, Solit DB (2014). Realizing the potential of plasma genotyping in an age of genotype-directed therapies. J Natl Cancer Inst.

[45] Jogo T, Nakamura Y, Shitara K, Bando H, Yasui H, Esaki T (2021). Circulating tumor DNA analysis detects FGFR2 amplification and concurrent genomic alterations associated with FGFR inhibitor efficacy in advanced gastric cancer. Clin Cancer Res.

[46] Shen SY, Burgener JM, Bratman SV, De Carvalho DD (2019). Preparation of cfMeDIP-seq libraries for methylome profiling of plasma cell-free DNA. Nat Protoc.

[47] Nuzzo PV, Berchuck JE, Korthauer K, Spisak S, Nassar AH, Abou Alaiwi S (2020). Detection of renal cell carcinoma using plasma and urine cell-free DNA methylomes. Nat Med.

[48] Xu RH, Wei W, Krawczyk M, Wang W, Luo H, Flagg K (2017). Circulating tumour DNA methylation markers for diagnosis and prognosis of hepatocellular carcinoma. Nat Mater.

[49] Luo H, Zhao Q, Wei W, Zheng L, Yi S, Li G (2020). Circulating tumor DNA methylation profiles enable early diagnosis, prognosis prediction, and screening for colorectal cancer. Sci Transl Med.

[50] Kim K, Shin DG, Park MK, Baik SH, Kim TH, Kim S (2014). Circulating cell-free DNA as a promising biomarker in patients with gastric cancer: diagnostic validity and significant reduction of cfDNA after surgical resection. Ann Surg Treat Res.

[51] Zhong Y, Fan Q, Zhou Z, Wang Y, He K, Lu J (2020). Plasma cfDNA as a potential biomarker to evaluate the efficacy of chemotherapy in gastric cancer. Cancer Manag Res.

[52] Kandimalla R, Xu J, Link A, Matsuyama T, Yamamura K, Parker MI (2021). EpiPanGI Dx: a cell-free DNA methylation fingerprint for the early detection of gastrointestinal cancers. Clin Cancer Res.

[53] Ren J, Lu P, Zhou X, Liao Y, Liu X, Li J (2022). Genome-scale methylation analysis of circulating cell-free DNA in gastric cancer patients. Clin Chem.

[54] Ling ZQ, Lv P, Lu XX, Yu JL, Han J, Ying LS (2013). Circulating methylated XAF1 DNA indicates poor prognosis for gastric cancer. PLoS One.

[55] Pimson C, Ekalaksananan T, Pientong C, Promthet S, Putthanachote N, Suwanrungruang K (2016). Aberrant methylation of PCDH10 and RASSF1A genes in blood samples for non-invasive diagnosis and prognostic assessment of gastric cancer. PeerJ.

[56] Hideura E, Suehiro Y, Nishikawa J, Shuto T, Fujimura H, Ito S (2020). Blood free-circulating DNA testing of methylated RUNX3 is useful for diagnosing early gastric cancer. Cancers.

[57] Alarcón MA, Olivares W, Córdova-Delgado M, Muñoz-Medel M, de Mayo T, Carrasco-Aviño G (2020). The Reprimo-like gene is an epigenetic-mediated tumor suppressor and a candidate biomarker for the non-invasive detection of gastric cancer. Int J Mol Sci.

[58] Yan H, Chen W, Ge K, Mao X, Li X, Liu W (2021). Value of plasma methylated SFRP2 in prognosis of gastric cancer. Dig Dis Sci.

[59] Balgkouranidou I, Matthaios D, Karayiannakis A, Bolanaki H, Michailidis P, Xenidis N (2015). Prognostic role of APC and RASSF1A promoter methylation status in cell free circulating DNA of operable gastric cancer patients. Mutat Res.

[60] Balgkouranidou I, Karayiannakis A, Matthaios D, Bolanaki H, Tripsianis G, Tentes AA (2013). Assessment of SOX17 DNA methylation in cell free DNA from patients with operable gastric cancer. Association with prognostic variables and survival. Clin Chem Lab Med.

[61] Han J, Lv P, Yu JL, Wu YC, Zhu X, Hong LL (2014). Circulating methylated MINT2 promoter DNA is a potential poor prognostic factor in gastric cancer. Dig Dis Sci.

[62] Hu XY, Ling ZN, Hong LL, Yu QM, Li P, Ling ZQ (2021). Circulating methylated THBS1 DNAs as a novel marker for predicting peritoneal dissemination in gastric cancer. J Clin Lab Anal.

[63] Wan JCM, Massie C, Garcia-Corbacho J, Mouliere F, Brenton JD, Caldas C (2017). Liquid biopsies come of age: towards implementation of circulating tumour DNA. Nat Rev Cancer.

[64] Yang J, Gong Y, Lam VK, Shi Y, Guan Y, Zhang Y (2020). Deep sequencing of circulating tumor DNA detects molecular residual disease and predicts recurrence in gastric cancer. Cell Death Dis.

[65] Ko K, Kananazawa Y, Yamada T, Kakinuma D, Matsuno K, Ando F (2021). Methylation status and long-fragment cell-free DNA are prognostic biomarkers for gastric cancer. Cancer Med.

[66] Openshaw MR, Mohamed AA, Ottolini B, Fernandez-Garcia D, Richards CJ, Page K (2020). Longitudinal monitoring of circulating tumour DNA improves prognostication and relapse detection in gastroesophageal adenocarcinoma. Br J Cancer.

[67] Varkalaite G, Forster M, Franke A, Kupcinskas J, Skieceviciene J (2021). Liquid biopsy in gastric cancer: analysis of somatic cancer tissue mutations in plasma cell-free DNA for predicting disease state and patient survival. Clin Transl Gastroenterol.

[68] Maron SB, Joshi SS, Lomnicki S, Oliwa T, Landron S, Johnson J (2018). Circulating tumor DNA (ctDNA) landscape and prognostic implications in advanced gastroesophageal adenocarcinoma (GEC). J Clin Oncol.

[69] Li J, Li Z, Ding Y, Xu Y, Zhu X, Cao N (2021). TP53 mutation and MET amplification in circulating tumor DNA analysis predict disease progression in patients with advanced gastric cancer. PeerJ.

[70] Karamitrousis EI, Balgkouranidou I, Xenidis N, Amarantidis K, Biziota E, Koukaki T (2021). Prognostic role of RASSF1A, SOX17 and Wif-1 promoter methylation status in cell-free DNA of advanced gastric cancer patients. Technol Cancer Res Treat.

[71] Yu JL, Lv P, Han J, Zhu X, Hong LL, Zhu WY (2014). Methylated TIMP-3 DNA in body fluids is an independent prognostic factor for gastric cancer. Arch Pathol Lab Med.

[72] Li J, Jiang W, Wei J, Zhang J, Cai L, Luo M (2020). Patient specific circulating tumor DNA fingerprints to monitor treatment response across multiple tumors. J Transl Med.

[73] Kim ST, Cristescu R, Bass AJ, Kim KM, Odegaard JI, Kim K (2018). Comprehensive molecular characterization of clinical responses to PD-1 inhibition in metastatic gastric cancer. Nat Med.

[74] Shin HM, Kim G, Kim S, Sim JH, Choi J, Kim M (2021). Chromatin accessibility of circulating CD8(+) T cells predicts treatment response to PD-1 blockade in patients with gastric cancer. Nat Commun.

[75] Chen Z, Zhang C, Zhang M, Li B, Niu Y, Chen L (2019). Chromosomal instability of circulating tumor DNA reflect therapeutic responses in advanced gastric cancer. Cell Death Dis.

[76] Jin Y, Chen DL, Wang F, Yang CP, Chen XX, You JQ (2020). The predicting role of circulating tumor DNA landscape in gastric cancer patients treated with immune checkpoint inhibitors. Mol Cancer.

[77] Kwak EL, Ahronian LG, Siravegna G, Mussolin B, Borger DR, Godfrey JT (2015). Molecular heterogeneity and receptor coamplification drive resistance to targeted therapy in MET-amplified esophagogastric cancer. Cancer Discov.

[78] Kim ST, Banks KC, Pectasides E, Kim SY, Kim K, Lanman RB (2018). Impact of genomic alterations on lapatinib treatment outcome and cell-free genomic landscape during HER2 therapy in HER2+ gastric cancer patients. Ann Oncol.

[79] Wu H, Fu M, Liu J, Chong W, Fang Z, Du F (2021). The role and application of small extracellular vesicles in gastric cancer. Mol Cancer.

[80] Pegtel DM, Gould SJ (2019). Exosomes. Annu Rev Biochem.

[81] Xie Y, Dang W, Zhang S, Yue W, Yang L, Zhai X (2019). The role of exosomal noncoding RNAs in cancer. Mol Cancer.

[82] Yan LX, Huang XF, Shao Q, Huang MY, Deng L, Wu QL (2008). MicroRNA miR-21 overexpression in human breast cancer is associated with advanced clinical stage, lymph node metastasis and patient poor prognosis. Rna.

[83] Sun J, Zhang Z, Bao S, Yan C, Hou P, Wu N (2020). Identification of tumor immune infiltration-associated lncRNAs for improving prognosis and immunotherapy response of patients with non-small cell lung cancer. J Immunother Cancer.

[84] Roy S, Kanda M, Nomura S, Zhu Z, Toiyama Y, Taketomi A (2022). Diagnostic efficacy of circular RNAs as noninvasive, liquid biopsy biomarkers for early detection of gastric cancer. Mol Cancer.

[85] Kahroba H, Samadi N, Mostafazadeh M, Hejazi MS, Sadeghi MR, Hashemzadeh S (2022). Evaluating the presence of deregulated tumoral onco-microRNAs in serum-derived exosomes of gastric cancer patients as noninvasive diagnostic biomarkers. Bioimpacts.

[86] Fan Y, Che X, Qu J, Hou K, Wen T, Li Z (2019). Exosomal PD-L1 retains immunosuppressive activity and is associated with gastric cancer prognosis. Ann Surg Oncol.

[87] In ‘t Veld S, Wurdinger T (2019). Tumor-educated platelets. Blood.

[88] Calverley DC, Phang TL, Choudhury QG, Gao B, Oton AB, Weyant MJ (2010). Significant downregulation of platelet gene expression in metastatic lung cancer. Clin Transl Sci.

[89] Nilsson RJ, Balaj L, Hulleman E, van Rijn S, Pegtel DM, Walraven M (2011). Blood platelets contain tumor-derived RNA biomarkers. Blood.

[90] Best MG, Sol N, Kooi I, Tannous J, Westerman BA, Rustenburg F (2015). RNA-Seq of tumor-educated platelets enables blood-based pan-cancer, multiclass, and molecular pathway cancer diagnostics. Cancer Cell.

[91] Siravegna G, Marsoni S, Siena S, Bardelli A (2017). Integrating liquid biopsies into the management of cancer. Nat Rev Clin Oncol.

[92] Millholland JM, Li S, Fernandez CA, Shuber AP (2012). Detection of low frequency FGFR3 mutations in the urine of bladder cancer patients using next-generation deep sequencing. Res Rep Urol.

[93] Wang Y, Springer S, Mulvey CL, Silliman N, Schaefer J, Sausen M (2015). Detection of somatic mutations and HPV in the saliva and plasma of patients with head and neck squamous cell carcinomas. Sci Transl Med.

[94] Pan W, Gu W, Nagpal S, Gephart MH, Quake SR (2015). Brain tumor mutations detected in cerebral spinal fluid. Clin Chem.

[95] Hoshino I (2021). The usefulness of microRNA in urine and saliva as a biomarker of gastroenterological cancer. Int J Clin Oncol.

[96] Li F, Yoshizawa JM, Kim KM, Kanjanapangka J, Grogan TR, Wang X (2018). Discovery and validation of salivary extracellular RNA biomarkers for noninvasive detection of gastric cancer. Clin Chem.

[97] Cui L, Zhang X, Ye G, Zheng T, Song H, Deng H (2013). Gastric juice MicroRNAs as potential biomarkers for the screening of gastric cancer. Cancer.

[98] Yu X, Luo L, Wu Y, Yu X, Liu Y, Yu X (2013). Gastric juice miR-129 as a potential biomarker for screening gastric cancer. Med Oncol.

[99] Shao Y, Ye M, Jiang X, Sun W, Ding X, Liu Z (2014). Gastric juice long noncoding RNA used as a tumor marker for screening gastric cancer. Cancer.

[100] Watanabe Y, Kim HS, Castoro RJ, Chung W, Estecio MR, Kondo K (2009). Sensitive and specific detection of early gastric cancer with DNA methylation analysis of gastric washes. Gastroenterology.

[101] Iwasaki H, Shimura T, Yamada T, Okuda Y, Natsume M, Kitagawa M (2019). A novel urinary microRNA biomarker panel for detecting gastric cancer. J Gastroenterol.

[102] Kao HW, Pan CY, Lai CH, Wu CW, Fang WL, Huang KH (2017). Urine miR-21-5p as a potential non-invasive biomarker for gastric cancer. Oncotarget.

[103] Hu L, Liu J, Shimada H, Ito M, Sugimoto K, Hiwasa T (2022). Serum Anti-BRAT1 is a common molecular biomarker for gastrointestinal cancers and atherosclerosis. Front Oncol.

